# Investigating the Effect of Zn Ferrite Nanoparticles on the Thermomechanical, Dielectric and Magnetic Properties of Polymer Nanocomposites

**DOI:** 10.3390/ma12183015

**Published:** 2019-09-17

**Authors:** Aikaterini Sanida, Sotirios G. Stavropoulos, Thanassis Speliotis, Georgios C. Psarras

**Affiliations:** 1 Smart Materials & Nanodielectrics Laboratory, Department of Materials Science, School of Natural Sciences, University of Patras, 26504 Patras, Greece; ksanida@upatras.gr (A.S.); stavropoulos@upatras.gr (S.G.S.); 2 Institute of Nanoscience and Nanotechnology, NCSR “Demokritos”, Aghia Paraskevi, 15310 Athens, Greece; t.speliotis@inn.demokritos.gr

**Keywords:** zinc ferrite, superparamagnetic, polymer nanocomposites, dielectric relaxations

## Abstract

In this study nanocomposites consisting of an epoxy resin and ceramic zinc ferrite nanoparticles have been successfully developed and investigated morphologically and structurally by means of scanning electron microscopy (SEM) images and X-ray diffraction (XRD) spectra. The thermal properties of the nanocomposites were studied via differential scanning calorimetry (DSC) and thermogravimetric analysis (TGA). The thermomechanical characterization of the fabricated nanocomposites was studied via dynamic mechanical analysis (DMA) and the magneto-dielectric response was assessed by means of a broadband dielectric spectroscopy (BDS) and by employing a superconducting quantum interference device (SQUID) magnetometer. Data analysis demonstrates that the incorporation of nanoinclusions into the matrix improves both the thermomechanical and the dielectric properties of the systems, as indicated by the increase of the storage modulus, the real part of dielectric permittivity and conductivity values with filler content, while at the same time induces magnetic properties into the matrix. Zinc ferrite nanoparticles and their respective nanocomposites exhibit superparamagnetic behavior at room temperature. Three relaxations were recorded in the dielectric spectra of all systems; originating from the filler and the polymer matrix, namely interfacial polarization, glass to rubber transition of the polymer matrix and the reorientation of small polar side groups of the polymer chain.

## 1. Introduction

Ultrafine nanosized particles of magnetic materials exhibit unique magnetic properties, in some cases remarkably different from those of their bulk counterparts [1]. These magnetic properties make magnetic nanoparticles suitable for many technological applications including magnetic data storage, ferrofluid, medical imaging, drug targeting and catalysis [2,3,4,5,6,7]. 

Nano ferrites with spinel structure type with the general formula of MFe_2_O_4_ (M = Ni^2+^, Mn^2+^ and Zn^2+^) are very interesting materials and attract enhanced attention due to their moderate saturation magnetization, high coercivity, high electrical resistivity and low eddy current losses, for applications like microwave devices, sensors, isolators, data storage, circulators, phase shifters, gyrators and information delivery devices [2,3,4,8].

Bulk zinc ferrite has a typical normal spinel structure with Zn ions in the tetrahedral or A sites and Fe ions in the octahedral or B sites. ZnFe_2_O_4_ was therefore initially regarded as an antiferromagnetic material with paramagnetic behavior at room temperature. However, outstanding changes have been observed in the magnetic properties of nanosized zinc ferrite particles, driving the currently increasing amount of research in ZnFe_2_O_4_ nanoparticles [7,9,10,11]. 

Polymer nanocomposites incorporating inorganic nanomaterials with advanced physicochemical properties receive extensive attention due to their unique advantages, which include ease processing and forming, thermomechanical stability, high dielectric breakdown strength and low cost [12,13,14]. Their electromagnetic properties can be altered by embedding suitable reinforcing inclusions and their dielectric and magnetic response can be tuned by controlling the type and the amount of filler content [15,16,17,18,19,20,21]. These unique properties make polymer nanocomposites, especially those filled with magnetic inclusions, suitable for advanced applications in various fields such as chemical sensors, magnetic recording materials, molecular electronic, electric catalysis, electro-magnetic interference (EMI) shielding, rechargeable battery, microwave absorption materials and corrosion protection coatings [2,3,4,5,6,22,23,24]. The vast majority of studies in the field investigate the properties of either composites containing non-stoichiometric zinc ferrite nanoparticles, where part of Zn is substituted by metallic ions like Ni or Co, or zinc ferrite incorporated in a conductive polymer matrix like PANI or PP [25,26,27,28,29,30,31,32]. In this study pure stoichiometric zinc ferrite nanoparticles have been incorporated in an insulating epoxy matrix, targeting to study the multifunctional behavior of the produced nanocomposites by assessing their thermal, thermomechanical, electrical and magnetic properties. By these means the next generation of polymer nanocomposites is expected to respond autonomously to as many external stimuli as possible and perform multiple functions exploiting their intrinsic properties, establishing thus their multifunctional performance. Epoxy resins are extensively used for the production of advanced composites in the fields of automotive, aerospace and electronics, mostly due to their high stiffness and good adhesion with the inclusions [33,34,35,36,37].

## 2. Materials and Methods

A series of ZnFe_2_O_4_ nanocomposites with various amounts of filler content were fabricated. The polymer matrix comprised of a two-component low viscosity epoxy resin (Epoxol 2004 A) along with its hardener (Epoxol 2004 B), provided by Neotex SA, Athens, Greece. ZnFe_2_O_4_ nanoparticles with size less than 100 nm, provided by Sigma-Aldrich, Saint Louis, MO, USA, were employed as filler. For the fabrication of the nanocomposites the prepolymer was mixed with the hardener at 2:1 w/w mixing ratio according to the supplier’s data sheet, followed by the incorporation of the nanoparticles into the mixture while stirring in a sonicator bath for 10 min. Afterwards, the mixture was poured into silicon molds with different geometries suitable for each characterization technique. Curing took place for seven days at room temperature and the post-curing treatment followed at 100 °C for 4 h. The fabricated concentrations were (0, 1, 3, 5, 10, 15 and 20 parts per hundred resin per weight (phr)). Nanocomposites with higher filler content were developed especially for the magnetic measurements (30, 40 and 50 phr).

The morphology of the nanocomposites was studied by scanning electron microscopy (SEM) using an EVO MA-10 apparatus provided by Carl Zeiss. Nanocomposites’ structure was identified by X-Ray diffraction (XRD) spectra that were obtained via Siemens Z500 diffractometer, using Cu-Ka (λ = 1.54056 Å, 40 kV, 30 mA) in a wide range of Bragg angles 20°–90°.

The thermal stability of the nanocomposites was assessed by thermogravimetric analysis (TGA) in the temperature range from 30 to 700 °C with 10 °C/min heating rate, using a TA Q500 device provided by TA instruments. The samples were placed in a platinum sample boat and the measurement was conducted under nitrogen atmosphere. The thermal properties of the nanocomposites were further investigated via differential scanning calorimetry (DSC), in the temperature range from 20 to 100 °C with 5 °C/min heating rate, using a TA Q200 device also provided by TA instruments. For the DSC tests, samples were placed in an aluminum crucible, while an empty aluminum crucible was serving as reference. The thermomechanical characterization was conducted by dynamic mechanical analysis (DMA) from room temperature to 100 °C, with a temperature rate of 5 °C/min and frequency of 1 Hz, using DMA Q800 by TA Instruments, in the 3-point bending configuration using rectangular samples (length = 50 mm, width = 13 mm and height = 3 mm).

The dielectric response of the nanocomposites was examined by means of broadband dielectric spectroscopy (BDS) using an Alpha-N Frequency Response Analyzer and a Novotherm System to control frequency and temperature, respectively. The measurements were recorded in the frequency range of 10^−1^–10^7^ Hz and temperature range of 30–160 °C, with a 5 °C temperature step, performing isothermal frequency scans with the amplitude of the time varying voltage being 1 V. The specimens (discs with diameter = 36 mm and height = 2 mm) were placed inside the dielectric cell BDS 1200. All equipment and software were provided by Novocontrol Technologies. 

The magnetic characterization was performed by a superconducting quantum interference device (SQUID) magnetometer by Quantum Design. The hysteresis loops were obtained at room temperature and maximum applied magnetic field of 50 kOe, while the zero field cooled and field cooled (ZFC/FC) procedures were conducted for a small magnetic field of 100 Oe and temperatures from 5 to 300 K. The specimens had a cylinder shape with diameter = 6 mm and height = 10 mm.

## 3. Results

**Figure 1:**
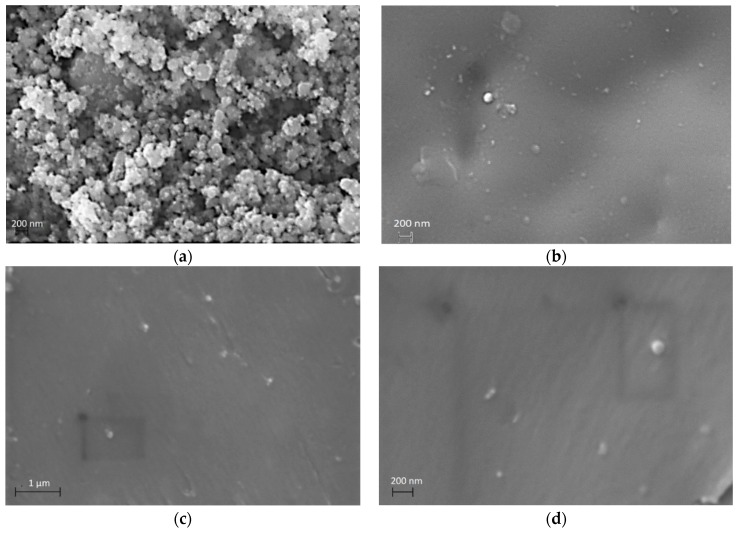
SEM images for (**a**) ZnFe_2_O_4_ nanoparticles and the nanocomposites with 5 phr filler content. (**b**) Surface; (**c**) cross-section (low magnification) and (**d**) cross-section (high magnification).

The quality of the nanocomposites was checked by SEM images. Representative SEM images as depicted in Figure 1a shows the morphology of the employed nanoparticles prior to their integration in the polymer matrix. Figure 1b,c provides evidence for the absence of bubbles, cracks or voids that could have a detrimental effect on the nanocomposites’ properties. Moreover, it seems that fine nanodispersions were accomplished and extensive agglomeration was avoided, even in specimens with high filler content. 

**Figure 2:**
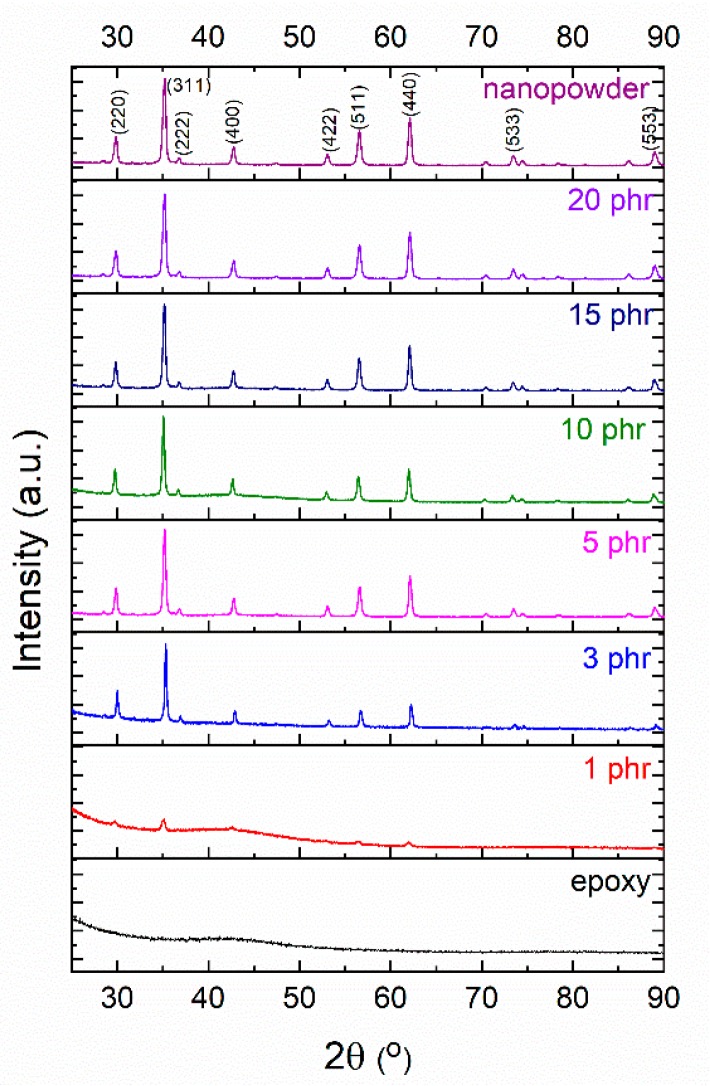
XRD spectra of the ZnFe_2_O_4_ nanopowder and its respective nanocomposites.

The major recorded peaks (220), (311), (400), (511) and (440) in the XRD spectra of the ZnFe_2_O_4_ nanopowder and the nanocomposites, as depicted in Figure 2, coincide in both position and intensity with the cubic, spinel structure with the Fd-3 m space group and lattice parameter a = 8.4411 Å, according to the International Centre for Diffraction Data [38].

**Figure 3:**
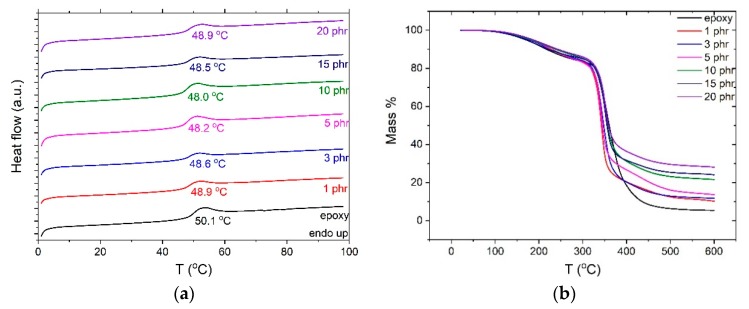
(**a**) DSC and (**b**) TGA thermographs for all examined systems.

DSC thermographs depicted in Figure 3a revealed an endothermic step-like transition of all systems, attributed to the glass to rubber transition of the polymer matrix. At the point of inflection of the transition, the characteristic glass transition temperature (T_g_) was defined by using suitable software provided by TA instruments. The variation of the glass to rubber transition temperature provides indications for the interactions occurring inside the nanocomposites [15,19]. All nanocomposites exhibit lower values of the glass transition temperature than the neat epoxy. Decreased values of T_g_ imply indirectly stronger interactions between the nanoparticles than between macromolecules and nanoinclusions. The addition of nanoparticles disrupts the crosslinking of the polymer network, thus increasing the free volume and enhancing the chain flexibility [39]. At higher filler concentrations, the excess number of nanoparticles applies spatial restrictions to macromolecules, obstructing thus the cooperative segmental mobility of the polymer chains and yielding to a slight increase of the glass to rubber transition temperature of the systems [19]. No exotherm reactions or other thermal events were recorded at the thermographs of all systems indicating the complete curing of the nanocomposites. 

The addition of nanoparticles affects the thermal properties of the nanocomposites by intervening with the crosslinking procedure and the formation of an interfacial area between the filler and the polymer matrix. The thermal stability of the nanocomposites was studied using thermogravimetric analysis at 10 °C/min heating rate under inert nitrogen atmosphere. The TGA curves of all systems are reported in Figure 3b. All nanocomposites and the pure epoxy exhibit two stages of weight loss. The first step took place approximately from 150 to 250 °C, where nanocomposites did not lose significant mass and the process could be attributed to the breaking of unreacted epoxy rings or the presence of impurity traces apart from the cured epoxy. In addition to humidity that could be filler-bounded, other low-mass compounds such as residual solvent, additives or contaminants might be present in polymers [40,41]. The incorporation of nanoparticles seems to shift the first step to higher temperatures, thus enhancing the thermal stability of the nanocomposites, as indicated by the temperature corresponding to 5% initial mass loss. The shift is more evident for the nanocomposites with higher filler content. The second stage took place between 300 and 400 °C with a maximum decomposition temperature higher than 330 °C, where the occurred significant weight loss corresponds to the decomposition of the epoxy matrix. The decomposition temperature was determined by the point of inflection of the degradation curves and is considered as the temperature of the maximum reaction (weight loss) rate. In all cases, nanocomposites exhibit lower degradation temperatures compared to the matrix (333 °C) although it increases with filler concentration. The values of char residue confirm the successful fabrication of the nanocomposites.

**Figure 4:**
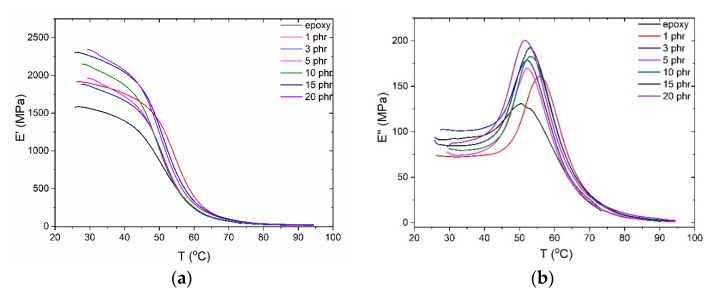
(**a**) Storage modulus and (**b**) loss modulus as a function of temperature, for all nanocomposites with ZnFe_2_O_4_ content.

The incorporation of the ceramic reinforcing nanoparticles enhances the thermomechanical properties of the nanocomposites due to their intrinsic stiffness and rigidness. As shown in Figure 4 the storage moduli increase monotonously, in general, with filler content, up to 50% for the highest filler content system, with the exception of the nanocomposite with 1 phr filler content, which records higher value of *E*’ than the 3 phr sample. The increment of *E*′ values, with reinforcing phase content, is attributed to the effective load transfer from the polymer matrix to the filler, resulting from the good dispersion and adhesion of filler in the matrix [42]. A steep decrease in the storage modulus values signifies the transition of the polymer matrix from the glassy to the rubbery state, where the nanocomposites lose their load retention capacity. Such transitions are represented by the formation of peaks in the loss modulus spectra. The slight shift of the peak to higher temperatures is an indication of good adhesion between the filler and the polymer matrix regardless of filler concentration. The unexpected higher temperature of the peak corresponding to the sample with 1 phr filler content signify a higher value of T_g_, related to obstructions to the synergetic relaxation of macromolecular chains. At low filler content the distance between nanoparticles is relatively large and their mutual interactions weak, thus the macromolecules-nanoparticles interactions prevail, leading subsequently to an induced stiffening of the sample, as expressed in Figure 4a. This situation becomes different at higher filler contents, since nanoinclusions are close to each other and their interactions become dominant [15,19]. At this point it should be noted that DMA examines the overall performance of the specimens, while DSC is a rather local technique since it tests only a small part of a specimen. Thus, any discrepancies occurring between the results from these two techniques could be attributed to this fact.

**Figure 5:**
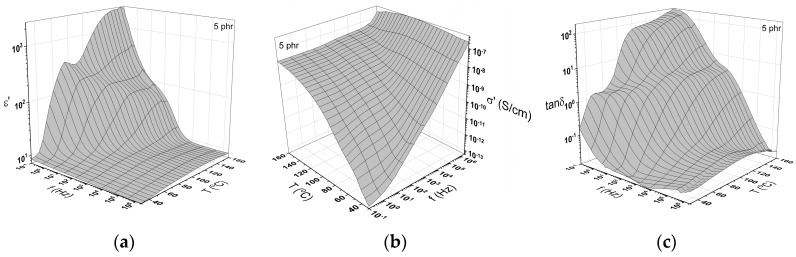
The variation of the: (**a**) Real part dielectric permittivity; (**b**) AC conductivity and (**c**) loss tangent as a function of frequency and temperature for the nanocomposite with 5 phr filler content.

A representative illustration of the dielectric response of all examined systems is depicted in Figure 5 in the form of 3D plots of (a) the real part of dielectric permittivity, (b) AC conductivity and (c) loss tangent as a function of frequency and temperature for the nanocomposite with 5 phr filler content. The real part of dielectric permittivity attains high values at low frequencies and high temperatures due to the thermal agitation that facilitates the alignment of the dipoles with the field. At higher frequencies, ε’ diminishes rapidly, since the large induced dipoles and permanent ones fail to follow the alternation of the externally applied field. A step like transition at intermediate frequencies and temperatures is attributed to the glass to rubber transition of the polymer matrix (α-relaxation). 

As shown in Figure 5b, AC conductivity increases with both temperature and frequency. The effect of temperature on the σ_AC_ values is more evident in the low frequency area signifying a thermally activated conduction mechanism. At intermediate frequencies and temperatures, the formation of a shoulder is related to dielectric relaxations, possibly the α-relaxation process.

Three distinct relaxations processes were recorded in the plots of the loss tangent as a function of frequency and temperature. These relaxation mechanisms were ascribed to the presence of the filler and the polymer matrix. At low frequencies and high temperatures, the process of interfacial polarization (IP), or Maxwell–Wagner–Sillars (MWS) effect, is recorded, due to the accumulation of free charges at the interface between the matrix and filler. At intermediate frequencies and temperatures, another loss peak is formed, ascribed to α-relaxation or in other words the glass to rubber transition of the polymer matrix, and at high frequencies, β-relaxation is observed, which is attributed to the reorientation of small polar side groups of the polymer chain.

## 4. Discussion

**Figure 6:**
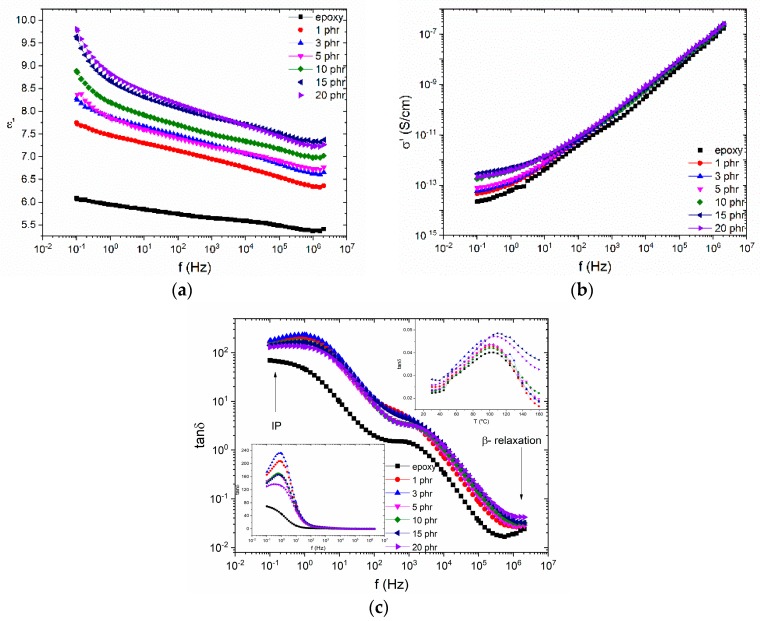
Comparative plots of (**a**) the real part of dielectric permittivity; (**b**) the AC conductivity, at 30 °C and the (**c**) loss tangent, at 160 °C, as a function of frequency, for the ZnFe_2_O_4_ examined systems. Inset (left down corner) depicts the variation of loss tangent versus frequency in a semi-logarithmic representation, for the same data, while the inset (right up corner) describes the loss tangent versus temperature, at 10^6^ Hz.

The influence of the zinc ferrite nanoparticles on the dielectric properties of the nanocomposites is demonstrated in Figure 6. The real part of the dielectric permittivity increases with filler content at the whole frequency and temperature range, due to the higher ε’ values of the ceramic nanofiller. This is more evident in the low frequency area, due to the interfacial polarization. The incorporation of nanoparticles has the same influence on the AC conductivity as well, since zinc ferrite is a semiconductor with a narrow band gap (1.9 eV). At high frequencies σ_AC_ attains high values because localized charge carriers hope between adjacent conductive sites, while at low frequencies they are forced to migrate to larger distances in confined conductive sites, addressing high potential barriers because of the presence of the insulating matrix. As it can be seen in Figure 6b, σ_AC_ seems to follow the exponential equation also known as the AC universality low, as expressed in Equation (1):

(1) $$\sigma_{\mathrm{AC}}\left(\omega \right)\;={\;\sigma }_{\mathrm{DC}}\;+\;A{\left(\omega \right)}^{s}$$

where σ_DC_ is the dc conductivity (ω = 0) and A, s are parameters depending on the temperature and filler content. 

All three recorded relaxations are presented in the plot of loss tangent versus frequency at 160 °C. α-relaxation is recorded at intermediate frequencies. Inset diagrams elucidate the effect of filler on interfacial polarization and β-relaxation at low and high frequencies, respectively. 

**Figure 7:**
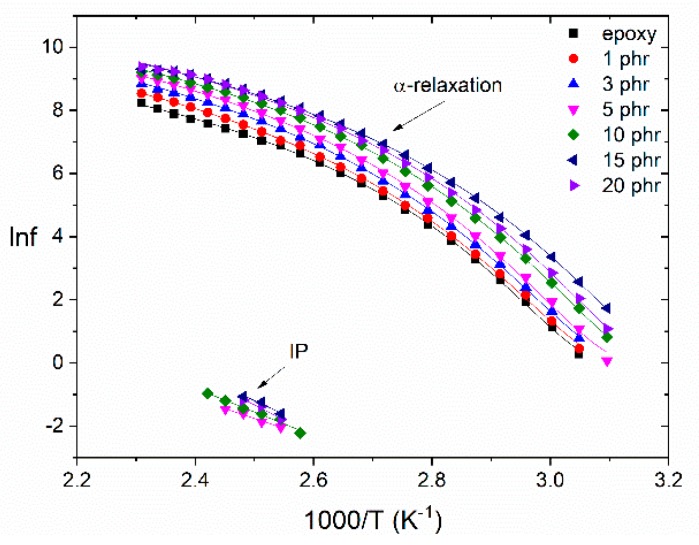
Loss peak position as a function of the reciprocal temperature for the IP and α-relaxation processes, for all the examined systems.

The investigation of the relaxation dynamics could provide an insight on the effect of filler loading and the interactions that occur in the polymer network at a molecular level. The dependence of the loss peak frequency on the temperature for all systems is depicted in Figure 7. The β-relaxation process is missing from this study due to limited number of loss peaks available for a reliable fitting. The temperature dependence of the IP mechanism is described by an Arrhenius type equation, as expressed in Equation (2):

(2) $$f_{max}\;=\;f_{0}e^{-\;\frac{E_{A}}{k_{B}T}}$$

where *f_max_* is the loss peak frequency, *f*_0_ a pre-exponential factor, *E_A_* is the relaxation’s activation energy, *k_B_* is the Boltzmann’s constant and *T* is the temperature. The temperature dependence of the α-relaxation process follows the VFTH (Vogel-Fulcher-Tammann-Hesse) equation, expressed by Equation (3):

(3) $$f\;=\;f_{0}e^{-\;\frac{Β{Τ}_{V}}{Τ-{Τ}_{V}}}$$

where *f*_0_ is a pre-exponential factor, *B* is a measure of the activation energy of the mechanism and *T_V_* the Vogel temperature or ideal glass transition temperature. All fitted parameters are listed in Table 1, as well as the values of activation energy for the IP process.

The large number of formed dipoles at the interface between the filler and the polymer matrix requires augmented thermal agitation for the activation of the IP mechanism, resulting in increasing values of activation energy. The activation energy of the IP process attains higher values with filler concentration, indicating the increasing heterogeneity of the systems. Notably, the increasing heterogeneity at high filler content is confirmed by the limited number of loss peaks for the nanocomposites with low filler content and the complete absence of IP for the neat epoxy. All nanocomposites exhibited lower *T_V_* values than the neat polymer matrix. This is an indirect indication of strong attractive interactions between the nanoparticles, which result in enhanced polymer chain flexibility. At the highest filler content, the excess number of nanoparticles obstruct the cooperative segmental mobility, thus, increasing the respective *T_V_* value. An analogous behavior was observed for parameter *B* indicating the facilitation of the glass to rubber transition with filler content up to the 15 phr specimen.

**Figure 8:**
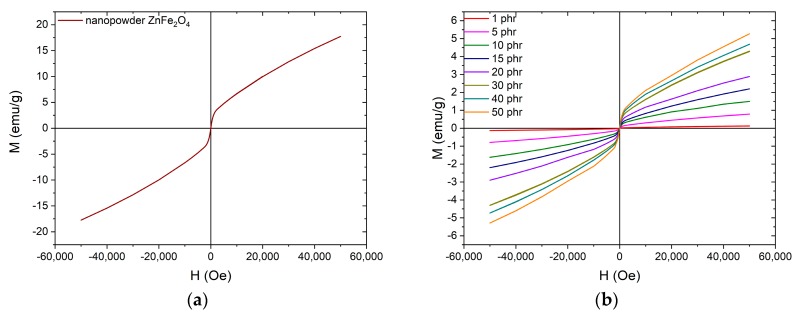
Magnetic hysteresis loops for the: (**a**) ZnFe_2_O_4_ nanopowder and (**b**) nanocomposites with varying ZnFe_2_O_4_ filler content.

The magnetic hysteresis loops for the nanopowder and the nanocomposites are presented in Figure 8. The magnetic behavior of both the nanopowder and the nanocomposites is characterized by the lack of coercivity and remanence magnetization. The latter combined with the difficulty of the magnetization to reach saturation even at high external fields is an indication that the nanoparticles are in the superparamagnetic state at room temperature. Nanocomposites’ magnetization increases with filler content since the zinc ferrite nanoparticles induce magnetic properties to the non-magnetic polymer matrix.

**Figure 9:**
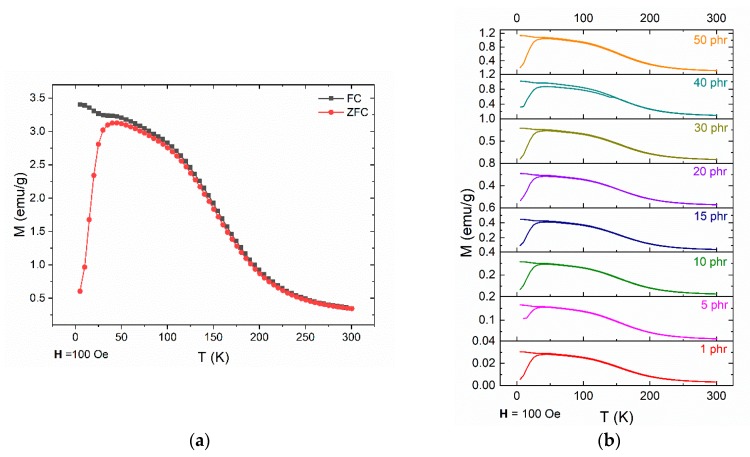
The ZFC and FC magnetization curves for (**a**) ZnFe_2_O_4_ nanopowder and (**b**) nanocomposites with varying ZnFe_2_O_4_ filler content.

In order to further investigate the magnetic properties of the nanocomposites especially at low temperatures, zero-field cooling (ZFC) and field cooling (FC) measurements were carried out. Figure 9 presents the temperature dependence of magnetization at an applied magnetic field of 100 Oe. In all cases, there is a formation of a peak at the ZFC curve, at approximately 43 ± 1 K. Below this temperature, also known as the blocking temperature (*T_B_*) the system is blocked, as expected for superparamagnetic nanoparticles. The ZFC and FC curves don’t merge with each other, providing indication of magnetic irreversibility, up to *T_irr_* ≈ 100 K. The difference between *T_B_* and *T_irr_* values demonstrate the existence of particles’ size distribution, which unblock at higher temperatures.

## 5. Conclusions

Zinc ferrite nanoparticles were incorporated into an epoxy thermoset matrix and their influence on the thermomechanical, dielectric and magnetic properties of the fabricated nanocomposites was investigated. The incorporation of the nanoparticles enhances both the thermomechanical and the dielectric response of the nanocomposites as demonstrated by the increasing values of the storage modulus, the real part of dielectric permittivity and AC conductivity with filler concentration. Three different relaxation mechanisms were identified in the dielectric spectra of all zinc ferrite nanocomposites. From the faster to the slower one, were attributed to: β-relaxation, due to the rearrangement of small, polar side groups of the polymer chain, glass to rubber transition of the polymer matrix (α- relaxation) and interfacial polarization (IP) due to accumulation of free charges at the interface between ZnFe_2_O_4_ nanoparticles and the polymer matrix. Zinc ferrite nanoparticles generate magnetic properties into the non-magnetic polymer matrix and as a result magnetization increases with filler content. All nanocomposites and the ceramic nanopowder exhibit superparamagnetic behavior at room temperature, as suggested by the lack of coercivity and remanence magnetization, with blocking temperature at approximately 43 K.

## Figures and Tables

**Table 1:** Specimens’ concentration in phr (parts per hundred per resin per weight), activation energy values and fitted parameters for all examined systems.

ZnFe_2_O_4_ Nanocomposites	IP	α-relaxation	
	*E_A_* (eV)	*T_V_* (K)	*B*
neat epoxy	-	315.9	17.5
1 phr	-	313.3	19.3
3 phr	-	311.6	19.4
5 phr	0.549	311.2	18.6
10 phr	0.649	307.7	18.0
15 phr	0.748	303.0	17.6
20 phr	0.832	306.1	18.0
